# Clinical Overlapping in Autoinflammatory Diseases: The Role of Gene Duplication

**DOI:** 10.3389/fimmu.2017.00392

**Published:** 2017-04-05

**Authors:** Paola Galozzi, Leonardo Punzi, Paolo Sfriso

**Affiliations:** ^1^Rheumatology Unit, Department of Medicine DIMED, University of Padova, Padova, Italy

**Keywords:** autoinflammatory diseases, NOD2, nod-like receptor protein 3, NLRP12, gene duplication, identical mutations

Most cases of autoinflammatory diseases (AIDs) are characterized by attacks of systemic inflammation resulting in recurrent fever, acute arthritis, and increased acute phase proteins. Frequent symptoms are also skin rash, myalgia, and bone deformations. The appropriate diagnosis of an AID can be challenging due to the overlapping symptoms or the presence of comorbidities, as autoimmune diseases. The discovery of the first autoinflammatory gene, *MEFV*, responsible for familial Mediterranean fever has dramatically increased the understanding of the pathophysiology of AIDs, shedding light on the associated defective signaling pathways (1). Since then, more than 20 autoinflammatory genes have been discovered, even though their clinical significance is often unclear. Moreover, an increasing number of reports describe cases bearing two mutations in a dominant-inherited AID, or patients with more than two mutations that segregate in a recessive AID, or mutated gene clusters. All these findings confuse the clinician leading to a delayed diagnosis or less accurate and relevant treatments.

The concept of autoinflammation originally emerged from the context of the monogenic hereditary periodic fever syndrome. Nowadays, it is worldwide accepted the hypothesis that AIDs are distributed along a continuum involving mutation type, number of genes, and environmental factors, ranging from monogenic to multifactorial conditions (2). An increasing number of studies support the involvement of sequence variants of gene responsible for Mendelian AIDs in multifactorial conditions (3). An example should be the Crohn’s disease, one of the first investigated complex diseases in which *NOD2* gene [responsible for Blau syndrome (BS)] was identified as a susceptibility gene. More recently, Jéru and colleagues describe the pathogenicity of a mutation affecting the same residue in TNFR1 in both Mendelian TRAPS syndrome and multifactorial inflammatory conditions (early arthritis, AA amyloidosis in juvenile idiopathic arthritis, multiple sclerosis, as examples) (4).

Thus, not only a single gene could be involved in various phenotypes but also many different genes could be entailed in overlapping phenotypes.

Consistent with this hypothesis, we propose that evolutionary gene duplication can have a causative role in AIDs development. Gene duplication is an important mechanism through which new genetic material is generated during molecular evolution. It can result from unequal crossing over, retroposition, or chromosomal/genome duplication (5). Duplication increases buffering capacity of genomes or species in adapting to changing environments, offering a selective advantage over a long-time scale. Functional redundancy resulting from gene duplication was reported to be a common phenomenon in biological systems by Podder and Ghosh (6). The assessment of the differences between monogenic and polygenic disease genes revealed that monogenic disease genes are functionally more buffered by their duplicates than polygenic ones. It seems akin to the current puzzling genetic situation of the AIDs.

A computational study by Wong and co-authors (7) observed that almost 50% of the disease proteins in the Ensembl database have duplicates associated with disease, suggesting that gene duplication is a significant phenomenon to the expansion of disease-related protein family. This reinforces the concept that sequence similarity to known disease proteins may also be a significant factor that contributes to disease propensity and implies common origins and mechanisms to many diseases.

We have focused our attention on the nucleotide-binding domain, leucine-rich repeat (LRR) (NLR)-containing family, important components of the innate immune system. Only a few members of this family, including the cytosolic pattern recognition receptors NOD2, nod-like receptor protein 3 (NLRP3), and NLRP12, have been analyzed extensively. These NLRs are known to have a pivotal role in the innate immune response toward microbial invasion and are identfied as responsible for clinical distinct AIDs, which shares pathophysiological characteristics (8).

Over the last decade, several mutations have been identified in *NOD2* gene, encoding for the intracellular sensor of bacterial cell wall components (NOD2) that triggers the activation of NF-κB and MAPK pathways. NOD2 has a typical three-domain structure composed of two N-terminal effector regions consisting of a caspase recruitment domain, a centrally located domain NACHT that is critical for protein activation, and nine C-terminal LRRs that sense the pathogens. Mutations on LRRs are known to confer susceptibility to Crohn’s disease, a chronic inflammatory bowel disorder, whereas mutations in the central NACHT domain are associated with a granulomatous disease, named BS. BS is a rare autosomal dominant AID clinically characterized by granulomatous recurrent uveitis, dermatitis, and symmetric arthritis (9). The most commonly observed BS-related mutations are high-penetrance missense substitutions affecting the highly evolutionary conserved Arginine residue at position 334 (p.R334W or p.R334Q).

*NLRP3* gene encodes a protein named cryopyrin or NLRP3, that comprise a N-terminal nucleotide-binding domain named pyrin, a central regulatory region NACHT and a C-terminal LRR domain. NLRP3, along with caspase-1 and an adaptor protein called ASC, was identified as a component of a cytoplasmic complex named inflammasome. NALP3 inflammasome is activated by a variety of factors, acting as a general detector of cell stress from pathogenic infection and intrinsic metabolic compounds. This inflammasome fulfills several cellular functions, from the activation of appropriate inflammatory processes through interleukin (IL)-1β to the activation of bacterial clearance processes.

A group of autoinflammatory disorders called cryopyrin-associated periodic syndromes or CAPS results from mutations associated to the NALP3 inflammasome (10). CAPS encompass three distinct autoinflammatory conditions, including familial cold autoinflammatory syndrome (FCAS), Muckle–Wells syndrome (MWS), and neonatal onset multisystemic inflammatory disease/chronic infantile neurological cutaneous articular syndrome (NOMID/CINCA). These conditions form a spectrum of diseases of increasing severity, from the mildest FCAS to the most severe (NOMID/CINCA). The symptoms of CAPS include recurrent fever, urticarial-like rash, arthralgia and/or arthritis, amyloidosis, and ocular involvement.

Interestingly, NLRP12 displays sequence and structure similarity to NLRP3 and plays a crucial role in immune system mechanisms against pathogenic agents (11). As CAPS-like disorders, NLRP12-associated disease can be induced by generalized exposure to cold and is characterized by recurrent fever episodes accompanied by skin rash, lymphadenopathy, and abdominal pain.

At protein structural level, the NLR proteins bear some similarities, as a centrally located NACHT domain and C-terminal LRR domains. A multiple sequence alignment reported by Albrect and colleagues have indicated a conserved structural core of the NACHT domain residues and reported an identical mutation at analogous position in NLRP3 and NOD2 (12). The mutations p.R260W in NLRP3 and p.R334W in NOD2 are described in literature as associated with FCAS/MWS and BS, respectively. The replacement of the positively charged Arginine with an hydrophobic Tryptophan results at protein level in a different environment that may impair the folding and the function of the NACHT domain. The p.R260W mutation is considered a frequent or unambiguous CAPS-linked mutation and has been shown *in vitro* to provoke spontaneous activation of the NLRP3 inflammasome (13). According to previous descriptions, the p.R260W mutation is associated with exanthematous rash, joint symptoms, and sight impairment, while fever is unusual. The position 334 in NOD2 protein instead is considered a hot spot for mutations since the majority of described BS-mutations involves this evolutionary conserved region (9). Clinical manifestations varied even among familial cases with p.R334W, but it tended to cause more obvious visual impairment.

We personally notice other identical mutations located at the same alignment position (Figure 1A). The mutations p.D303N in NLRP3 and p.D383N in NOD2 presented all clinical criteria of the associated diseases, CINCA/MWS, and BS, respectively (14, 15). The substitution of the negatively charged Aspartic acid with a non-charged Asparagine may alter the folding in the NACHT domain. The mutations p.E304K in NLRP3 and p.E383K in NOD2 are known to be associated with CINCA and BS, respectively. The substitution of the negatively charged Glutamate with a positively charged Lysine may alter a fine-tuned regulatory mechanism linked to ATP hydrolysis in the NACHT domain. The p.E304K variation has been predicted as disease-causing mutation, based on position in the protein, molecular nature, and evolutionary conservation of the residue changed (16). It is associated with rash, persistent arthritis, and uveitis but without fever. The p.E383K mutation is known to impair the function of NOD2 but without a primary mediation of IL-1β and other pro-inflammatory cytokines (17). Clinical manifestations associated with p.E383K are papulonodular skin eruption, mild arthritis of the hands and feet, and severe chronic bilateral uveitis, evolving in panuveitis.

**Figure 1:**
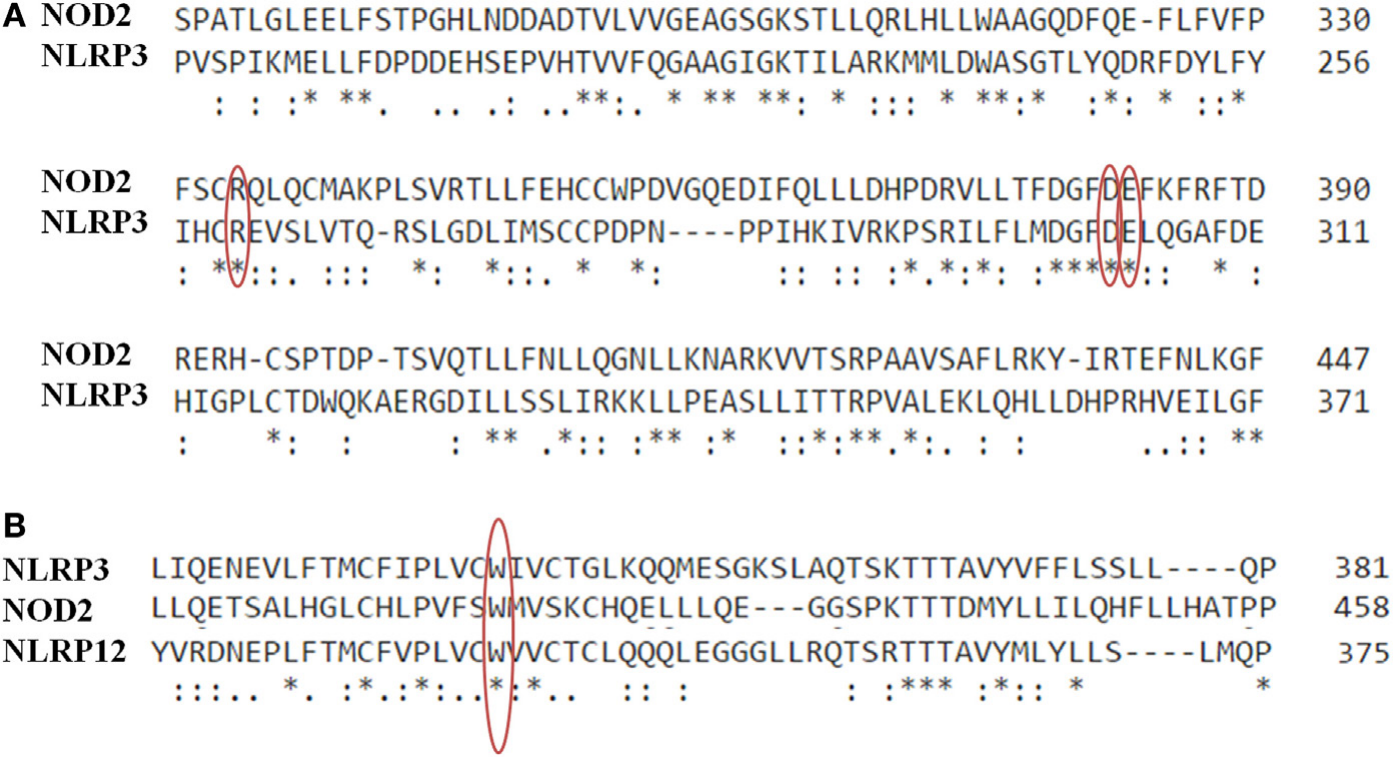
**(A)** Multiple sequence alignment of NOD2 (NP_071445.1) and nod-like receptor protein 3 (NLRP3) (NP_001230062.1) in the central portion of NACHT domains by means of CLUSTAL Omega (EMBL-EBI). Alignment with NLRP12 (NP_653288.1) is also presented in **(B)**. The asterisk (*) indicates positions with a single, fully conserved residue. The colon (:) indicates a strong conservation of residues with strongly similar properties, while the period (.) marks residues with weakly similar properties. Red circles highlight the mutations at the same position in the alignment (p.R334W/p.R260W; p.D382N/p.D303N; p.E383K/p.E304K; p.W408X/p.W414L/p.W490L).

Based on *in silico* analysis reported by Jéru et al. (11), we have observed a mutation affecting the same residue at analogous position in the structural core of the NACHT domain of NLRP3, NLRP12, and NOD2 (Figure 1B). The mutations p.W408X in NLRP12, p.W414L in NLRP3, and p.W490L in NOD2 are known to be associated with FCAS, MKW, and BS, respectively. The substitution of the aromatic Tryptophan with a aliphatic Lysine may alter the formation of hydrogen bonds in the NACHT domain. The stop-gain mutation (p.W408X) may instead lead to protein dysfunction and, therefore, contribute to disease pathogenesis. This nonsense mutation in NLRP12 is found associated with recurrent fever and skin urticaria due to cold conditions (18). The other variations have been reported as AID-causing mutations in Infevers database (19).

Identical mutations at analogous conserved sequence positions are extremely improbable to occur by chance. It is also improbable that a mutation affecting the same residue in a highly conserved sequence regions of a protein family is a fortuitous event. In fact, there is also a good identity percentage between NOD2 and NLRP3 sequences (26.72% complete identity and 18.30% of strong similarity) and NLRP3 and NLRP12 sequences (49.89% complete identity). Common clinical manifestations are also reported for the identical mutations, i.e., the absence of fever, the increased ocular involvement, and skin rash in the variations presented above. These findings suggest a close relationship between BS, CAPS, and NLRP12-associated disease, not only because both disorders affect the innate immune system but also because of common evolution.

Investigation of the mechanisms that generate duplicate gene copies and the subsequent dynamics among gene duplicates represent a potential area for shedding light on evolutionary aspects related to interactions among all AIDs. From our point of view, the mechanism of gene duplication could help to explain the overlap of clinical symptoms and the great genetic heterogeneity in AIDs. We expect that the exponential increase in genomic data and rapid advances in molecular genetic technology will provide more evidence to support our hypothesis.

## Funding Information

This work was supported by Institutional funds (DOR1640251/16) from the University of Padova.

## Author Contributions

GP contributed to the conception and to draft the work. Both PL and SP also contributed to revise it critically.

## Conflict of Interest Statement

The authors declare that the research was conducted in the absence of any commercial or financial relationships that could be construed as a potential conflict of interest.
